# The GET Complex Mediates Insertion of Tail-Anchored Proteins into the ER Membrane

**DOI:** 10.1016/j.cell.2008.06.025

**Published:** 2008-08-22

**Authors:** Maya Schuldiner, Jutta Metz, Volker Schmid, Vladimir Denic, Magdalena Rakwalska, Hans Dieter Schmitt, Blanche Schwappach, Jonathan S. Weissman

**Affiliations:** 1 Howard Hughes Medical Institute, Department of Cellular and Molecular Pharmacology, University of California, San Francisco, and California Institute for Quantitative Biosciences, San Francisco, CA 94158, USA; 2 Zentrum für Molekulare Biologie der Universität Heidelberg (ZMBH), Im Neuenheimer Feld 282, Heidelberg D-69120, Germany; 3 Department of Molecular Biology, Max-Planck-Institute for Biophysical Chemistry, Göttingen D-37077, Germany

**Keywords:** CELLBIO, PROTEINS

## Abstract

Tail-anchored (TA) proteins, defined by the presence of a single C-terminal transmembrane domain (TMD), play critical roles throughout the secretory pathway and in mitochondria, yet the machinery responsible for their proper membrane insertion remains poorly characterized. Here we show that Get3, the yeast homolog of the TA-interacting factor Asna1/Trc40, specifically recognizes TMDs of TA proteins destined for the secretory pathway. Get3 recognition represents a key decision step, whose loss can lead to misinsertion of TA proteins into mitochondria. Get3-TA protein complexes are recruited for endoplasmic reticulum (ER) membrane insertion by the Get1/Get2 receptor. In vivo, the absence of Get1/Get2 leads to cytosolic aggregation of Get3-TA complexes and broad defects in TA protein biogenesis. In vitro reconstitution demonstrates that the Get proteins directly mediate insertion of newly synthesized TA proteins into ER membranes. Thus, the GET complex represents a critical mechanism for ensuring efficient and accurate targeting of TA proteins.

## Introduction

The biogenesis of transmembrane proteins presents the cell with several compounding challenges. Prior to membrane insertion, hydrophobic transmembrane domains (TMDs) are prone to aggregation, and the spontaneous insertion of TMDs across lipid bilayers, even when thermodynamically favored, can be slow. Moreover, proteins containing TMDs must find their correct target membrane for insertion among the different membrane-surrounded compartments present in eukaryotic cells. To face these challenges, cells have evolved diverse mechanisms for chaperoning membrane proteins, often from the earliest stages of their biosynthesis on the ribosome to their proper destinations. Such pathways have been the subject of intense investigations and include the signal recognition particle (SRP)/Sec61 translocon system that imports secretory pathway proteins into the endoplasmic reticulum (ER) (Egea et al., 2005; Rapoport et al., 1999; Wickner and Schekman, 2005) and the transport inner membrane/transport outer membrane (Tim/Tom) translocases that mediate insertion of transmembrane proteins into both mitochondrial membranes (Neupert, 1997; Pfanner and Meijer, 1997).

Far less is known about the machinery responsible for the insertion of an important class of proteins that are anchored to the lipid bilayer by a single TMD located near their C termini. This topological arrangement allows tail-anchored (TA) proteins to be tethered to internal membranes while presenting their functional N-terminal domains to the cytosol (Borgese et al., 2007; Wattenberg and Lithgow, 2001). TA proteins are found throughout the secretory pathway, in the nuclear envelope, peroxisomes, and mitochondria. Within the secretory pathway, TA proteins play diverse roles, such as enabling vesicular traffic (e.g., many of the SNAREs, which mediate fusion of secretory vesicles, are TA proteins [Beilharz et al., 2003]), aiding in protein translocation, and promoting folding or degradation of membrane proteins (Borgese et al., 2007; Wattenberg and Lithgow, 2001). Secretory pathway TA proteins are first inserted into the ER membrane, and are then sorted to their ultimate destination (Bulbarelli et al., 2002). In contrast, mitochondrial TA proteins are inserted directly into the mitochondrial membrane, where they facilitate mitochondrial fission, provide key components of the translocation machinery, and act in apoptosis (Borgese et al., 2007; Wattenberg and Lithgow, 2001). The membrane specificity of TA proteins is largely encoded in their TMDs and flanking regions (Egan et al., 1999). These signals, however, are not absolute, as some TA proteins, such as the mammalian oncoprotein Bcl2 (Krajewski et al., 1993; Lithgow et al., 1994), are found in both the mitochondria and the ER. Moreover, it is not well understood how targeting determinants in the TMDs are decoded by cellular machinery (Borgese et al., 2007).

Because of its position near the C terminus, the TMD of TA proteins is occluded by the ribosome until translation is completed. Thus, TA proteins cannot exploit the classic cotranslational SRP/Sec61 translocation mechanism used by most secretory pathway proteins (Yabal et al., 2003). Early studies with cell extracts indicated that some TA proteins, such as CytB5, could integrate into membranes without the assistance of specialized machinery (Brambillasca et al., 2006; Rachubinski et al., 1980). However, most TA proteins, such as the mammalian Sec61β and synaptobrevin, have more hydrophobic TMDs, rendering them reliant on an incompletely characterized, ATP-dependent mechanism (Abell et al., 2007; High and Abell, 2004; Stefanovic and Hegde, 2007; Favaloro et al., 2008).

Recently, biochemical studies identified the mammalian soluble ATPase, Asna1/TRC40, as part of a cytosolic complex that interacts with the newly synthesized TA protein, Sec61β, in vitro (Stefanovic and Hegde, 2007; Favaloro et al., 2008). This complex can then deliver Sec61β to the surface of ER-derived vesicles (microsomes), where upon it can undergo ATP-dependent membrane insertion. While these studies have provided critical molecular insights into the ATP-dependent biogenesis of TA proteins, they leave several important questions unaddressed. First, it is unclear how broad a role the Asna1/TRC40 system plays in vivo. Indeed, a recent report established that the cytosolic chaperone pair Hsc70/Hsp40 is sufficient to mediate efficient ATP-dependent insertion of Sec61β in vitro (Abell et al., 2007). Second, the identity of the proteins necessary for recruiting Asna1/TRC40 to the ER is unknown. Finally, it is unknown how cells ensure proper partitioning of TA proteins between the ER and mitochondria.

Based on a large-scale genetic interaction map of the secretory pathway, we previously suggested that three otherwise unassociated yeast proteins (Mdm39/Get1, Rmd7/Get2, and Arr4/Get3, the yeast homolog of Asna1/TRC40) cooperate to carry out a common function that strongly impacts on trafficking and, accordingly, named them Get1–3 (Golgi ER trafficking 1–3) (Schuldiner et al., 2005). In agreement with this idea, we and others have found that all three Get proteins physically associate (Auld et al., 2006; Ho et al., 2002; Schuldiner et al., 2005), and that loss of any of the *GET* genes leads to a pronounced Kar2 secretion phenotype, suggestive of a defect in retrograde Golgi to ER trafficking (Schuldiner et al., 2005). However, the full range of phenotypes that have now been reported for the respective *get* deletions are difficult to reconcile with an isolated defect in trafficking. These include mitochondrial dismorphogenesis (Dimmer et al., 2002) for *Δget1 (Δmdm39)*; defects in DNA replication or damage response (Zewail et al., 2003) and V-type ATPase dysfunction (Sambade et al., 2005) for *Δget2 (Δhur2/Δrmd7)*; sensitivity to toxic metal ions (Shen et al., 2003) and effects on protein degradation machinery (Auld et al., 2006) for *Δget3 (Δarr4)*; and defects in meiotic spore formation (Auld et al., 2006; Enyenihi and Saunders, 2003) for all deletions in *GET* genes. Thus, the underlying molecular function(s) of the Get proteins, and the extent to which they are working together to perform a single molecular role, remained unresolved.

Here we show, both in vivo and in vitro, that the GET complex is the machinery responsible for insertion of secretory pathway TA proteins into the ER membrane, and that the reduction in inserted TA proteins can, in turn, explain the wide array of phenotypes observed for deletions in the *GET* genes.

## Results

### Get1 and Get2 Form a Membrane Receptor for Get3 on the Face of the ER

**Figure 1:**
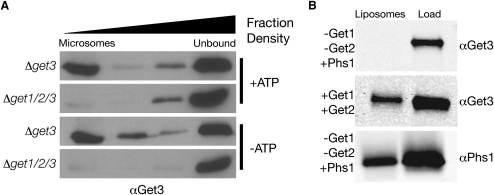
Get1 and Get2 Act as a Membrane Receptor for the Soluble Get3 (A) Western blots with αGet3 showing binding of recombinant Get3 ATPase to microsomes prepared from Δ*get3* or Δ*get1/2/3* strains in the presence or absence of ATP. Shown are Optiprep gradient fractions, which separate microsomes from unbound protein. (B) Western blots with αGet3 or αPhs1 showing binding of recombinant Get3 to proteoliposomes reconstituted with either Phs1 as a control protein (−Get1/−Get2+PHS1) or purified Get1-PC and Get2-HA (+Get1/+Get2). Shown are optiprep gradient fractions as above.

We began our functional analysis of the GET complex by exploring how Get1 and Get2 determine the subcellular localization of Get3 (for analysis of the physical and functional relationship between the Get proteins see Figures S1 and S2 available online). Earlier studies established that Get3, which, unlike Get1 and Get2, is not predicted to have TMDs, is found on the surface of the ER as well as in the cytosol. Moreover, in the absence of Get1 and/or Get2, Get3 loses its ER localization, and is found both in the cytosol as well as in poorly characterized punctate structures (Auld et al., 2006; Schuldiner et al., 2005). Here we reveal that, rather than being membrane vesicles, these punctate structures are in fact cytosolic detergent-insoluble aggregates (Figure S3). We further show, through in vitro experiments with microsomes and proteoliposomes containing Get1 and Get2, that the Get1/Get2 complex is directly responsible for recruiting Get3 to the ER membrane in an ATP-independent manner (Figure 1). This appears to be the primary role of Get1/2 complex, as, in the absence of Get3, there is no apparent additional cost to deleting Get1/2 (Auld et al., 2006; Schuldiner et al., 2005) (Figure S4). The fact that Get3 shuttles between the cytosol and the ER suggests that it may deliver substrates to the membrane. In the context of this model, the formation of aggregates and the exacerbated phenotype found in *Δget1/Δget2* cells (Auld et al., 2006; Schuldiner et al., 2005) (Figure S4) would be explained by disruption of the Get3 cycle, leading to sequestration of potential substrates.

### Get3 Binds the TA Protein Sed5 and Is Necessary for Its Membrane Targeting

**Figure 2:**
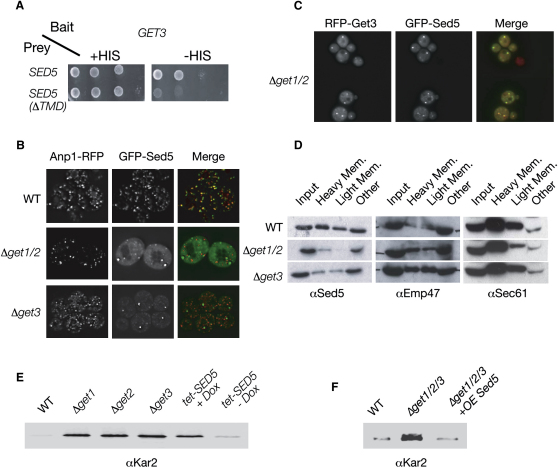
Get3 Binds to Sed5 and Is Important for Its Biogenesis In Vivo (A) Yeast two-hybrid assay with Get3 as bait and Sed5_197–340_ (the strongest hit from the Y2H screen) as prey (in the presence or absence of its TMD). The growth on medium lacking histidine (−HIS) is indicative of a physical interaction. (B) Fluorescence microscopy demonstrating a shift in the subcellular localization of GFP-Sed5 from Golgi in control (WT) strains to a partially cytosolic localization in a *Δget3* strain, and both cytosolic and few large puncta in *Δget1/2* strains. The GFP-Sed5 puncta in *get* mutants do not colocalize with the Golgi marker Anp1-RFP. (C) Fluorescence microscopy demonstrating colocalization of GFP-Sed5 and Get3-tdRFP in cytosolic aggregates that form in a *Δget1/2* background. (D) Western blots of cell fractionation experiments to determine levels of Sed5 in membrane fractions. Control (WT), *Δget1/2* or *Δget3* strains were divided into three fractions (Heavy Mem, Lighter Mem, and remainder of cellular proteins [Other]) and compared to input protein (Input) with Western blots immunostained against either Sed5 or the control Golgi transmembrane protein, Emp47, and ER transmembrane protein Sec61. (E) Western blots of secreted proteins with αKar2. Assay for Kar2 secretion was performed on a control strain (WT), mutants of the GET complex (*Δget1, Δget2, Δget3*), and on a yeast strain harboring a repressible allele of the essential TA protein Sed5 (tet-*SED5*), either in the presence (+Dox) or absence (−Dox) of the corepressor doxycycline. (F) Western blots of secreted proteins with αKar2. Assay for Kar2 secretion was performed on the triple mutant (*Δget1/2/3*) either alone or overexpressing *SED5* from a high copy plasmid (+ *OE SED5*), and compared to a control strain (WT).

To help identify factors that might be shuttled from the cytosol to the ER by the GET system, we performed a yeast two-hybrid (Y2H) screen for polypeptides that can interact with Get3. Y2H analysis, which reports on weak interactions occurring within the nucleus of assayed strains, is well suited for identifying Get3 binding proteins, as it can detect transient interactions that are independent of the presence of Get1 and Get2. We used yeast expressing Get3 as bait to screen a genomic library encoding prey proteins (James et al., 1996). Physical interactions caused activation of the Gal4-driven HIS3 reporter gene, allowing growth on plates lacking histidine. The strongest hit from the screen was a fragment of Sed5 (amino acid 197 to the C terminus) (Figure 2A), a TA protein that acts as a SNARE in vesicular traffic within the Golgi and between the Golgi and the ER (Hardwick and Pelham, 1992). The Get3-Sed5 interaction was dependent on the presence of the C-terminal TMD (Figure 2A).

We next examined whether Get3, as part of the GET complex, plays a role in recruiting newly synthesized Sed5 in the cytosol and inserting it into membranes. We visualized the subcellular localization of Sed5 with an N-terminal fusion protein with GFP (GFP-Sed5) (Weinberger et al., 2005). N-terminal GFP fusion was compatible with the correct targeting of Sed5 to the Golgi in control cells (Banfield et al., 1994; Weinberger et al., 2005) (Figure 2B). Deletion of Get3 led to a large pool of soluble protein and a corresponding decrease in Golgi-like puncta containing Sed5 (Figure 2B). In a *Δget1/Δget2* background, this defect was more pronounced; there was only modest Golgi staining and, instead, we observed cytosolic fluorescence and a few large punctate structures that were distinct from the Golgi, as visualized by Anp1-GFP staining (Figure 2B). Red fluorescent protein (RFP) fused to Get3 (Get3-tdRFP) and GFP-Sed5 colocalized in these punctate structures (Figure 2C). Thus, in the absence of the Get proteins, a substantial fraction of Sed5 remains in the cytosol. Consistent with this, subcellular fractionation experiments indicate that deletion of the *GET* genes leads to reduced levels of endogenous untagged Sed5 in membranes, while not interfering with membrane association of the Golgi protein Emp47 or the ER protein Sec61 (Figure 2D).

Decreased Sed5 SNARE activity in vesicles traveling between the Golgi and ER could slow down retrograde traffic and reduce the efficiency of cellular retrieval mechanisms of ER resident proteins (Hardwick and Pelham, 1992; Yamaguchi et al., 2002). We therefore tested whether reduced Sed5 function could explain the Kar2 secretion phenotype observed in the *get* mutants. Consistent with this hypothesis, lowering protein levels of the essential Sed5 protein, by using a repressible tetO_7_ promoter (Mnaimneh et al., 2004), caused Kar2 secretion at levels that were similar to those observed in deletions of GET complex members (Figure 2E). Moreover, overexpression of Sed5, presumably by allowing sufficient Sed5 to insert into membranes by alternate, potentially spontaneous TA-insertion pathways (see Discussion), suppressed the Kar2 secretion defect in a triple-deletion strain that has no GET complex members (Figure 2F). We therefore conclude that the GET complex plays a major role in the biogenesis of the TA protein Sed5. In addition, the Kar2 secretion phenotype of these cells could be explained by the reduced levels of Sed5 in the membranes of *get* deletion mutants.

### The GET Complex Plays a Broad Role in Insertion of TA Proteins into Membranes

**Figure 3:**
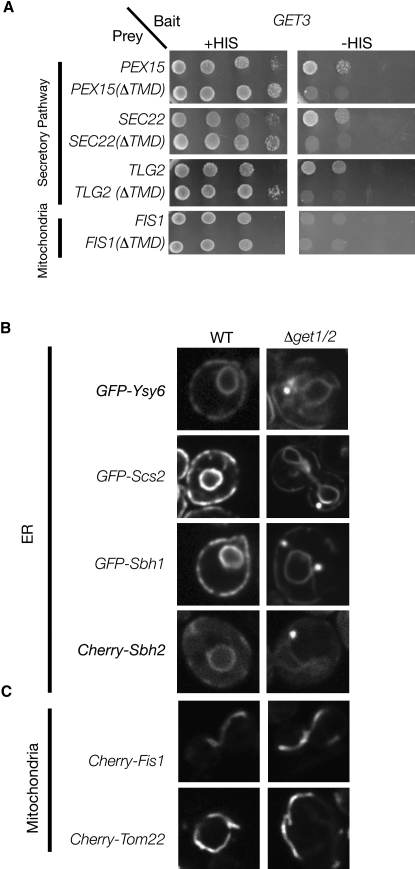
The GET Complex Affects the Biogenesis of a Wide Variety of TA Proteins (A) Y2H assay showing Get3 as bait and various TA proteins (in the presence or absence of their TMDs) as prey. The growth on medium lacking histidine (−HIS) is indicative of a physical interaction. (B) Fluorescence microscopy of control (WT) and *Δget1/2* strains expressing a broad variety of TA proteins. GFP-Scs2, GFP-Sbh1, and GFP-Ysy6 under a galactose-inducible (*GAL*) promoter. Cherry-Sbh2 was expressed from a plasmid under the constitutive *TEF2* promoter. (C) Fluorescence microscopy of control (WT) and *Δget1/2* strains expressing two mitochondrial TA proteins, Cherry-Fis1 and Cherry-Tom22, expressed from a plasmid under the constitutive *TEF2* promoter.

The role of the GET complex as a specific chaperone system for the TA protein Sed5 cannot explain the diversity of the phenotypes displayed by deletions of *GET* genes. The recent finding that the mammalian Get3 homolog Asna1 was involved in the insertion of in vitro synthesized Sec61β (Stefanovic and Hegde, 2007; Favaloro et al., 2008) suggests that the GET complex has a broader role in TA protein biogenesis. Consistent with this idea, by a directed Y2H approach, we detected physical interactions between Get3 and several additional secretory pathway TA proteins, including the SNAREs Tlg2 and Sec22 and the peroxisomal TA protein Pex15. These interactions, as observed for Sed5, were dependent on the presence of the C-terminal TMD (Figure 3A). Although these data suggest that Get3 can specifically recognize a range of C-terminally located TMDs, it appears to have some selectivity, as we could not detect physical interactions for the mitochondrial TA protein Fis1 (Figure 3A), which, like other mitochondrial TA proteins, has a shorter, more hydrophilic TMD than secretory pathway TA proteins (Beilharz et al., 2003; Borgese et al., 2001, 2007).

To test whether the observed physical interactions reflect an in vivo role for the GET complex in the biogenesis of secretory pathway TA proteins, we looked at the effect of loss of the Get1/Get2 receptor on the subcellular localization of a functionally diverse range of TA proteins. We focused predominantly on ER-localized TA proteins, as interpretation of effects on their localization is not complicated by trafficking defects seen in *get* mutant strains. Accordingly, we expressed N-terminal fluorescent protein fusions to Sbh1, Sbh2—the yeast homolog of Sec61β shown to interact with Asna1 (Stefanovic and Hegde, 2007)—Scs2, and Ysy6. As observed for Sed5, localization of these TA proteins was normal in control cells, but was altered in a *Δget1/Δget2* background (Figure 3B). During logarithmic growth, we could observe both the presence of large puncta (that colocalize with Get3) and also proper ER localization, (Figure S5). Following the diauxic shift, which occurs as cells exit log phase, we observed a more pronounced defect. In most cells, the majority of the protein was either cytosolic or in one or two large puncta that also contained Get3 (Figure S6). These defects are specific for TA proteins entering the secretory pathway, as the two mitochondrial TA proteins examined (Fis1 and Tom22) properly localized in both the control and a *Δget1/Δget2* background (Figure 3C).

### Loss of the GET Complex Leads to Mislocalization of a Subset of TA Proteins

**Figure 4:**
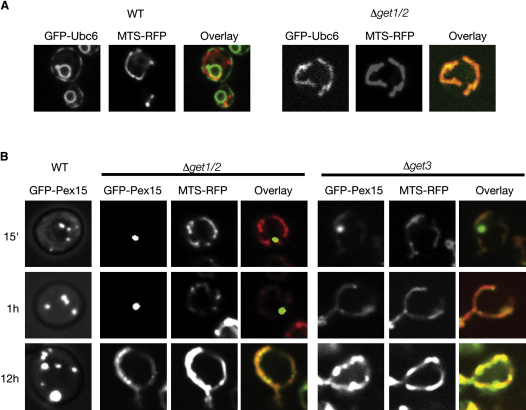
Role of GET Proteins in Creating Membrane Specificity (A) Fluorescence microscopy showing the localization of GFP-Ubc6 and mitochondrially targeted dsRED (MTS-RFP) in a control (WT) or *Δget1/2* strain. (B) Fluorescence microscopy of a time course monitoring the subcellular localizations of the peroxisomal TA protein GFP-Pex15 as well as dsRED targeted to the mitochondria (MTS-RFP) following induction of Pex15 from a galactose inducible promoter in a control (WT), *get1/2*, or *get3* strain.

The finding that Get3 is able to distinguish between TA proteins destined for the secretory pathway and those destined to mitochondria suggests that, in addition to increasing the efficiency of TA protein membrane insertion, the GET complex helps ensure that TA proteins accurately find their destination membrane. Consistent with this idea, we observed that, when overexpressed, a subset of secretory pathway TA proteins mislocalize to mitochondria in *Δget1/Δget2* strains (an example of the TA protein Ubc6 is given in Figure 4A). This effect was particularly pronounced for Pex15. In wild-type (WT) cells, this protein is thought to be first inserted into the ER, and then transported to the peroxisome via Pex19 (Elgersma et al., 1997; Hoepfner et al., 2005; Tam et al., 2005) (supported by data in Figure S7). However, in *Δget1/Δget2* cells, Pex15 initially formed cytosolic aggregates (Figure S8), but, after extended overexpression, began to appear in mitochondrial membranes (Figure 4B). This suggests that, once Pex15 saturated Get3, it could insert inappropriately into mitochondrial membranes. Indeed, we observed that, in the absence of Get3, this lag phase is shortened dramatically, and Pex15 is found in the mitochondria at much earlier time points (Figure 4B). Hence, in addition to increasing the efficiency of insertion of TA proteins, recognition by Get3 represents a key decision step in defining the membrane destination of a TA protein, thus overcoming the intrinsic potential for TA proteins to spontaneously insert into a wide range of membranes.

### Loss of TA Proteins Recapitulates the Pleiotropic GET Phenotypes

The diversity of TA protein functions suggests that the pleiotropic effects associated with loss of the GET complex might be a secondary consequence of TA protein mislocalization. To test this idea, we assembled a library of strains carrying mutant alleles for each of the predicted 55 yeast TA proteins (Beilharz et al., 2003) (including the six TA proteins localized to mitochondria), and plated these strains on various conditions for which we observed sensitivity in the *get* deletion strains. The library consisted of 43 deletion strains (Giaever et al., 2002) for nonessential TA proteins and 12 hypomorphic alleles of the essential ones. Hypomorphic alleles were made by using the decreased abundance by mRNA perturbation (DAmP) method (Schuldiner et al., 2005), which typically results in ∼5- to 10-fold decrease in levels of the endogenous protein.

**Figure 5:**
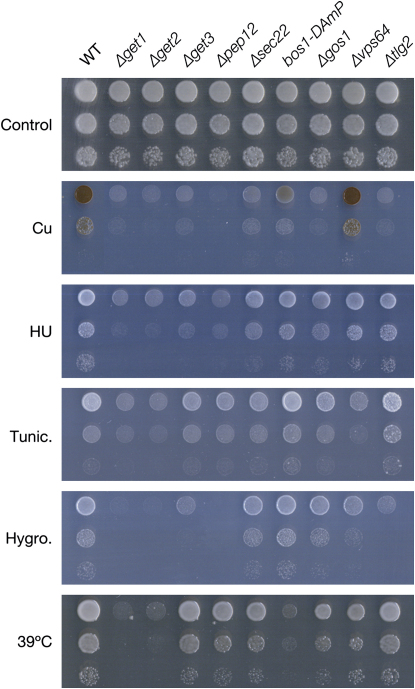
Reduced Levels of TA Proteins Can Explain the Diverse Array of GET Complex Phenotypes Serial dilutions in different conditions: SD + CuSO_4_ (Cu), SD + hydroxyurea (HU), SD + tunicamycin (Tunic.), SD + hygromycin (Hygro.), and YPD incubated at 39°C (39°C). Strains shown are: control cells (WT), *get* mutants, five TA protein deletion strains, and a strain carrying a hypomorphic allele (DAmP) of an essential TA protein. Copper sensitivity in the Δget3 strain is more pronounced in methionine prototrophic than auxotrophic cells. We used Δmet15 cells for this panel, resulting in a less sensitive phenotype compared with MET^+^ cells depicted in Figure S4.

We found that loss of a large number of TA proteins (*Δpep12*, *Δtlg2*, *Δsec22*, *Δvam3*, *Δscs2*, *Δsso2*, *Δgos1*, and *bos1-DAmP*) caused pronounced copper sensitivity (see Figure 5 for the most sensitive strains). This is consistent with an important role of the late secretory pathway in cellular copper homeostasis (Labbe and Thiele, 1999). More generally, individual TA protein mutant strains were sensitive to only a subset of the conditions. However, for every condition tested, we found a subset of TA protein deletions/depletions that fully recapitulate the sensitivities found in the *Δget1* and *Δget2* strain (Figure 5 shows the strains with strongest sensitivities). Thus, a broad defect in secretory pathway TA protein insertion could fully account for the diverse phenotypes observed upon loss of the Get1/Get2 receptor.

### In Vitro Evidence that the GET Complex Directly Mediates Insertion of Newly Synthesized TA Proteins into the ER membrane

The above studies establish that the Get proteins play a critical role in the biosynthesis and proper localization of a wide range of TA proteins. Given the pleiotropy of phenotypes displayed by the *get* deletants, we wished to assess whether the Get proteins are directly required for insertion of TA proteins into the ER membrane. We therefore developed an in vitro system for studying this process, which takes advantage of our ability to prepare cytosol extracts and microsomes from *get* mutant yeast strains.

**Figure 6:**
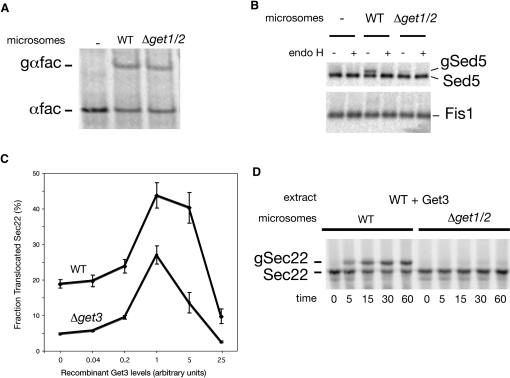
In Vitro Reconstitution of GET-Dependent Insertion of TA Proteins (A) Autoradiograph of in vitro-translated, ^35^S methionine-labeled, α-factor (αfac) following incubation in the presence of microsomes derived from WT or Δ*get1/2* strains. The position of untranslocated prepro-αfac and glycosylated, translocated pro-αfac (gαfac) are indicated. (B) Autoradiograph of in vitro translated, ^35^S methionine-labeled Sed5 and Fis1 following incubation with microsomes derived from WT or Δ*get1/2* strains. Prior to SDS-PAGE analysis, samples were immunoprecipitated with an anti-opsin antibody and then treated with EndoH, as indicated. The position of untranslocated Sed5 and Fis1 as well as glycosylated translocated Sed5 (gSed5) are indicated. (C) Graph representing the dose dependence of Sec22 translocation on addition of recombinant Get3 to either WT- or Δg*et3*-derived translation extracts. WT microsomes were added following translation, and the amount of glycosylated Sec22 relative to total Sec22 was calculated. Results from three independent experiments are shown; data are presented as mean ± SD. (D) Autoradiograph of in vitro-translated, ^35^S methionine labeled, Sec22 following translation in WT cytosol supplemented with optimal levels of Get3. Translocation was terminated at the indicated times following addition of microsomes derived from either WT or *Δget1/2* strains. The position of untranslocated Sec22 as well as glycosylated translocated Sec22 (gSec22) are indicated.

To monitor membrane insertion, we engineered a glycosylation site after the TA sequence of each substrate examined. Following translocation, this site is expected to gain access to the glycosylation machinery in the lumen of the ER (Borgese et al. [2001] and Figure S9) and, as such, serve as a proxy for translocation. Indeed, when we combined cytosol and microsomes from WT cells, we observed efficient translocation of preproalpha factor (a canonical Sec61 substrate) (Figure 6A) and of the three secretory pathway TA proteins that we tested: Sed5 (Figure 6B), Sec22 (Figures 6C and 6D), and Ysy6 (Figure S9). In contrast, we did not detect any translocation of the mitochondrial TA protein Fis1 (Figure 6B), indicating that our in vitro system faithfully recapitulates the target membrane specificity of TA protein insertion.

To evaluate the role of the Get proteins in TA protein insertion, we prepared extracts from a *Δget3* strain and microsomes from a *Δget1/2* strain. Strikingly, these mutant extracts and microsomes were defective for insertion of TA proteins (Figures 6B–6D and S9), while being fully proficient in supporting the translocation of preproalpha factor (Figure 6A and data not shown). Addition of recombinant Get3 to *Δget3* extracts during (Figure 6C), but not after (data not shown), translation allowed for robust insertion, thus demonstrating that this defect is a proximal consequence of not having Get3 in the in vitro system, and not due to altered cellular physiology in *Δget3* strains. Get3 also appears to be limiting in our WT extracts, as we saw enhanced translocation when recombinant Get3 was added (Figures 6C and S9). Critically, this Get3-mediated insertion is completely dependent on the presence of the Get1/2 complex in the microsomes (Figures 6D and S9), providing further evidence that these three proteins cooperate to carry out insertion of TA proteins. Taken together, these data establish that the GET system is directly responsible for mediating insertion of newly synthesized TA proteins into the ER membrane.

## Discussion

**Figure 7:**
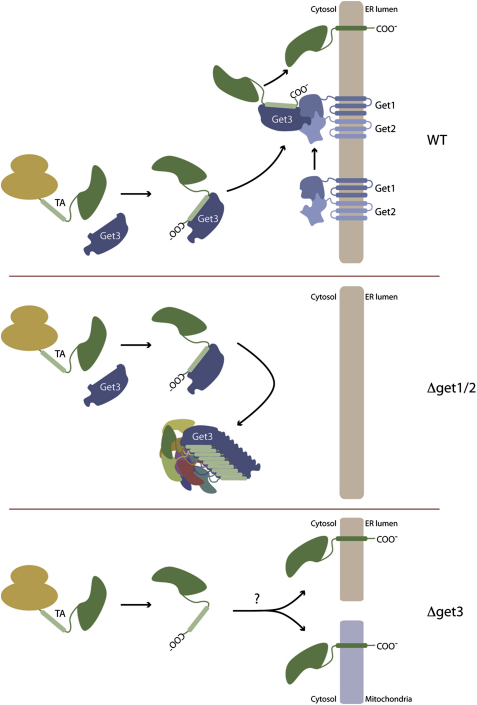
Schematic Model for GET Complex Function (Top) WT cells. Get3 recognizes newly synthesized, ER-destined TA proteins. The Get3-TA complexes dock onto the Get1/Get2 receptor. This allows insertion of TA proteins. (Middle) Cells lacking the receptor (*Δget1/2*). Get3-TA complexes fail to reach the ER and, instead, are sequestered in cytosolic aggregates. (Bottom) Cells lacking Get3 (*Δget3*). Newly synthesized TA proteins intended for the ER are no longer shuttled into the GET pathway. To varying degrees, depending on the TA proteins, they may use alternate ATP/GTP-dependant pathways or spontaneous routes for membrane insertion. This could lead to misinsertion into the mitochondria, inefficient insertion into the ER, or aggregation in the cytosol.

The present study defines a pathway by which cells ensure the efficient and accurate biogenesis of TA proteins destined for the secretory pathway. The soluble cytosolic ATPase, Get3, specifically queries newly synthesized proteins for the presence of C-terminally localized hydrophobic domains. Get1 and Get2 then serve as an ER membrane receptor, which recruits the Get3-TA complex, thereby promoting the proper insertion of TA proteins into the ER (Figure 7A). Once inserted, TA proteins can then be routed to their ultimate destination within the secretory pathway. In the absence of the heteromeric Get1/Get2 receptor, TA proteins bound to Get3 fail to reach ER membranes, and are instead trapped in large cytosolic aggregates (Figure 7B). This leads to a broad depletion of TA proteins, which in turn can account for the otherwise confusing array of phenotypes associated with loss of Get proteins. Binding to Get3 is also a decisive step in the insertion pathway, as in its absence, secretory pathway TA proteins may insert into mitochondrial membranes (Figure 7C).

The finding that the GET pathway is not essential for yeast viability provides in vivo support to in vitro studies that had suggested additional mechanisms by which TA proteins can find their destination membranes (Rabu and High, 2007). Nonetheless, several considerations suggest that the GET pathway is the major route used to target a broad range of TA proteins to the secretory pathway. First, our Y2H analysis indicates that Get3 can bind multiple secretory pathway TA proteins in a TMD-dependent manner. Second, for all secretory pathway TA proteins examined, the interaction with Get3 caused sequestration of the TA proteins into cellular aggregates in the absence of Get1 and Get2. This suggests that, when Get3 is present, most of the natural flux of TA proteins flows through the GET pathway. Indeed, yeast fail to grow when Get3 is overexpressed in the absence of Get1 and Get2 (Figure S4). Third, deletion of Get3, which would eliminate the GET pathway without actively preventing TA proteins from utilizing alternate pathways by trapping them in nonproductive Get3 complexes, still leads to diverse cellular defects. Finally, in vitro reconstitution experiments directly establish that Get3 cooperates with the Get1/2 complex in mediating the insertion of newly synthesized TA proteins. Thus the ability of cells to survive in the complete absence of the Get proteins may be analogous to the viability of yeast missing the SRP, which is made possible by the existence of alternate pathways for insertion of the numerous secreted and membrane-bound proteins that normally utilize this machinery (Ogg et al., 1992).

Possible alternate routes for TA protein biogenesis that have been suggested by in vitro studies include spontaneous insertion, which occurs efficiently for some TA proteins, such as CytB5 (Brambillasca et al., 2006). In addition, purified Hsc70/Hsp40 can promote the ATP-dependent (Abell et al., 2007) and SRP the GTP-dependent insertion of other TA proteins, such as Sec61β, (Abell et al., 2004). Such back-up systems, however, would lack the strong membrane specificity conferred by the ER localization of the Get1/2 complex, as well as the preferential binding of Get3 to TA proteins destined to the secretory pathway. The potential importance of such specificity is illustrated by the observation that some TA proteins, including Pex15 and Ubc6, mislocalize to the mitochondria when the GET system is impaired. This argues that, shortly after synthesis, Get3 competes with other factors (possibly Hsc70 and/or components that play an analogous role to Get3 in the targeting of mitochondrial TA proteins) for TMD binding, and that Get3 recognition commits the TA proteins to their subsequent insertion into ER membrane. It remains to be determined whether a dedicated protein machinery exists that ensures the accurate targeting of mitochondrial TA proteins, or whether the shorter, more hydrophilic nature of their TMDs prevents Get3 binding, thereby allowing for efficient, spontaneous insertion into the mitochondria.

The interaction between Get3 and a TA protein substrate may thus represent a critical and potentially regulated decision step for establishing the destination target of TA proteins. Regulation could globally alter Get3 function or specifically affect the interaction between Get3 and target TA proteins. Along these lines, we have recently found that the function of Get3 is modulated by its redox state (our unpublished data and Metz et al. [2006]). In addition, Get3 is transcriptionally upregulated under both cytosolic (Auld et al., 2006) and ER (Travers et al., 2000) stress conditions. It has also been found that many TA proteins are palmitoylated (Roth et al., 2006) or phosphorylated (such as for Sed5 [Weinberger et al., 2005]) on residues that are immediately adjacent to the TMD. Such modifications could modulate Get3 recognition by creating negatively charged flanking regions or by altering the hydrophobicity of the TMD, thereby enabling the coordinated regulation of subclasses of TA proteins and altering the physiology of the cell.

While the present studies focused on TA biogenesis in yeast, recent observations suggest that the GET pathway plays an essential role in TA biogenesis in higher eukaryotes. Biochemical studies revealed that the mammalian Get3 homolog, Asna1/TRC40, binds the TA protein, Sec61β, and facilitates its posttranslational insertion into ER membranes (Stefanovic and Hegde, 2007; Favaloro et al., 2008). An in vivo role of Asna1 in TA biosynthesis in metazoans is suggested by the impaired capacity for insulin secretion in *Caenorhabditis elegans* mutants of *asna1* (Kao et al., 2007). In light of our findings, an attractive hypothesis is that impaired insulin secretion results from compromised biogenesis of one or more of the SNARE TA proteins. The broader importance of the GET pathway is underscored by the finding that complete loss of *ASNA1* causes early embryonic lethality in mice (Mukhopadhyay et al., 2006) and arrested growth at the L1 stage in *C. elegans* (Kao et al., 2007). The molecular identity of the Get3 ER receptor in metazoans remains to be established. However, we find that Ysy6 translated in rabbit reticulocyte extracts inserts into yeast microsomes in a Get1/2-dependent manner, suggesting that the GET pathway is highly conserved (data not shown). Consistent with this, PSI-BLAST analysis identifies the WRB protein as an excellent and ubiquitously expressed candidate for a Get1 ortholog.

In summary, the GET complex in yeast and likely metazoans constitutes the major machinery necessary for membrane selective, and ATP-dependent insertion of TA proteins. This finding should now enable mechanistic studies to explore central questions, including how the GET system selects substrate and exploits ATP hydrolysis to overcome the energetic barriers to insertion of transmembrane proteins into lipid bilayers.

## Experimental Procedures

### Strains and Media

Due to a high rate of reversion, all deletions in *GET* genes were constructed by sporulating from a heterozygous diploid carrying deletions in all three genes (*his3Δ1/his3Δ1 leu2Δ0/leu2Δ0 LYS2/LYS2 MET15/met15Δ0 ura3Δ0/ura3Δ0 can1Δ::STE2pr-spHIS5/CAN1 lyp1Δ::STE3pr-LEU2/LYP1 cyh2/CYH2 GET1/Δget1::cgURA3 GET2/Δget2::NAT^r^ GET3/Δ::Kan^r^*). Following sporulation, single-, double-, and triple-deletion strains of the correct genotype were chosen. For Figure 5, all strains were chosen to be *Δmet15* to be isogenic with deletion strains taken from the Yeast Consortium Deletion Library (Giaever et al., 2002) or made by the DAmP method (Schuldiner et al., 2005). Deletion constructs used were pFA6-NAT and pFA6-Kan (Longtine et al., 1998) or pCG-URA (Kitada et al., 1995). Galactose (GAL) inducible strains were made on the same background as the deletions, only pFA6-Kan^r^-GALp or pFA6- Kan^r^-pGAL-GFP cassette was used (Longtine et al., 1998). C-terminally tagged Get3-GFP::His, Anp1-RFP::Kan^r^, and Pex3-RFP::Kan^r^ were taken from the whole genome GFP tag library (Huh et al., 2003). The tetO_7_-*SED5* strain was picked from the essential gene promoter shut-off collection (Mnaimneh et al., 2004). All N-terminal-tagged proteins with mCherry were created with a pFA6-based vector (Kind gift from David Breslow, University of California, San Francisco), carrying a URA3-TEF2 promoter-mCherry, and were integrated into the gene by one-step PCR-based homologous recombination, with appropriate primers that also introduced an N-terminal linker (GDGAGL) between the mCherry and the proteins. pRS315-GFPSed5 was a kind gift of Anne Spang (Weinberger et al., 2005). p416MET25-Get3-tdRFP was constructed by fusing the tdRFP (Campbell et al., 2002) open reading frame to the 3′ end of the GET3 open reading frame via an engineered NotI site coding for three alanines. For colocalization purposes, a mitochondrial targeting sequence containing RFP was used as a mitochondrial marker (kind gift of Jodi Nunnari, University of California, Davis). For the strong overexpression plasmid employed in Figure 1H, *SED5* was cloned into the 2 μm plasmid BFGIII under the control of its own promoter.

Plates used for drug sensitivity assays were: SD + 100 mM HU (Sigma), SD + 1μg/ml tunicamycin (Sigma), SD + 100 μg/ml hygromycin (Sigma), and SD + 1mM CuSO_4_. YPD plates were used for heat sensitivity assays at 39°C. For induction of the *GAL*, promoter cells were grown in YP + 2%Galactose.

### Microsome Binding Experiments

Microsomes were isolated from *Δget1/2/3* and *Δget3* yeast strains as previously described (Wuestehube and Schekman, 1992), then resuspended in reaction buffer (20 mM HEPES/KOH, pH 6.8, 5 mM MgAc_2_, 150 mM KAc, 250 mM sorbitol).

A volume of 10 μl of microsomes were mixed with 1 μl of 2 μM Get3 purified from *Escherichia coli* (Metz et al., 2006), 0.5 μl 100 mM glutathione (Sigma), 1.25 μl 100 mM batho cuproine disulfonic acid (BCS; SERVA), 1 μl 100 mM ATP (Sigma), and 14 μl of 2× ATPase buffer (200 mM HEPES/KOH, 20 mM MgCl_2_, 40% glycerol, pH 7.0), and were incubated at 30°C for 1 hr. After incubation, samples were immediately mixed with 490 μl of 50% Optiprep (PROGEN Biotechnik GmbH) solution in the reaction buffer, placed in 2 ml ultracentrifugation tubes, and overlayed with 1160 μl 40% Optiprep solution in reaction buffer, and, finally, with 450 μl of the reaction buffer. Samples were centrifuged at 166,000 × *g* for 3 hr at 4°C. After centrifugation, four fractions were collected: (1) 630 μl; (2) 430 μl; (3) 430 μl; (4) 640 μl. All fractions were precipitated with 50% TCA. Pellets were washed twice with 500 μl cold acetone and dried at 37°C for 1–5 min. Final pellets were resuspended in 1× SDS-PAGE sample buffer.

### Purification of GET Components

Get3 was purified as previously described (Metz et al., 2006). Epitope-tagged versions of Get1 and Get2 were copurified from yeast (see Experimental Procedures in the Supplemental Data).

### Liposome Binding Experiments

Proteoliposomes were prepared as described previously (Denic and Weissman, 2007) and incubated with recombinant Get3. as described above in Microsome Binding Experiments.

### Y2H System

The Y2H system was performed as previously described (Metz et al., 2006). For more details see Experimental Procedures in the Supplemental Data.

### Fluorescence Microscopy

For Figure 2C, microscopy was performed with a Leica DM IRE2 microscope (Leica Microsystems, Wetzlar, Germany). For Figure 2B, a DeltaVison restoration microscope was employed. Raw images were deconvolved with the additive algorithm of Softworx software. For live cell imaging, yeast were incubated in synthetic complete medium at room temperature. Fixed yeast cells were mounted in ProLong Gold antifade reagent with DAPI (Invitrogen). For Figures 3 and 4, microscopy was performed in the UCSF Nikon Imaging Center with a Yokogawa CSU-22 spinning disc confocal on a Nikon TE2000 microscope. For more detailed information see Experimental Procedures in the Supplemental Data.

### Crude Fractionation

OD_600_ units of 25–50 were harvested from log-phase cells growing in YPAD medium, washed once in water, and resuspended in 1 ml buffer (20 mM HEPES/KOH, pH 7.3, 100 mM KCl, 1 mM glutathione, complete protease inhibitors, phosphatase inhibitors [Roche], 1 mM EDTA, 1 mM EGTA, 3 mM BCS, 1 mM PMSF). Cells were broken by bead beating with 800 μl glass beads for 10 min. Homogenates were cleared at 2000 rpm in a microcentrifuge and the supernatant (input) was subjected to two sequential centrifugation steps (13,000 rpm in a microcentrifuge [Heavy membranes] and 40,000 rpm in a TLA45 rotor in a tabletop ultracentrifuge [Light membranes]). Pellets from both steps were resuspended in 250 μl (Heavy) or 50 μl (Light) of the same buffer as above. Equal protein concentrations of the collected fractions and the remaining supernatant (Other) were loaded, resolved by SDS-PAGE, transferred to a nitrocellulose membrane, and analyzed by immunoblotting with antisera against Sed5, Sec61, or Emp47.

### Kar2 Secretion Assays

Kar2 secretion assays were performed as previously described (Schuldiner et al., 2005). For more details see Experimental Procedures in the Supplemental Data.

### In Vitro Transcription

mRNAs were prepared with the mMessage mMachine kit (with cap analog, either SP6- or T7-driven, as appropriate) from Ambion. Alpha factor mRNA was transcribed from pDJ100 (Garcia et al., 1991). For other messages, template DNA was derived from PCR products amplified with a 5′ primer containing the T7 promoter/alpha factor 5′ UTR/kozak sequence/start codon and 5′ region of homology, and a 3′ primer containing, in antiparallel order, the 3′ region of homology preceding the stop codon/opsin tag/alpha factor 3′ UTR/polyA tail. Primer sequences are available upon request from the corresponding authors.

### In Vitro Translation and Translocation

Yeast translational extracts were prepared from cells grown to OD_600_ 1–2 in YPD. Cells were washed and resuspended in 1 ml of lysis buffer (100 mM KOAc, 2 mM Mg(OAc)_2_, 2 mM DTT, 20 mM HEPES-KOH, pH 7.4, complete protease inhibitors from Roche) for every 6 g of dry cell pellet. The cell slurry was frozen in liquid nitrogen and lysed by bead beating. The thawed lysates were spun in an SS34 rotor at 10,000 × *g* for 10 min. The low-speed supernatant was then spun in a TLA110 rotor at 49,000 rpm for 30 min. The high-speed supernatant was collected (avoiding the very top and bottom layers) and passed over a 5 × 5 ml (attached in series) HiTrap desalting column (GE Healthcare) equilibrated in lysis buffer with 14% glycerol. OD_280_ fractions >40 were collected, pooled, and stored as frozen aliquots at −80°C.

Prior to use, extracts were treated with micrococcal nuclease (Amersham or NEB) to remove any endogenous mRNAs, as described previously (Garcia et al., 1991).

Translation reactions contained 9.5 μl of nuclease-treated extract, 2.5 μl of 6× mix (132 mM HEPES-KOH, pH 7.4, 720 mM KOAc, 9 mM Mg(OAc)_2_, 4.5 mM ATP, 0.6 mM GTP, 150 mM creatine phosphate [Roche], 0.24 mM of each amino acid except methionine [Promega], 10.2 mM DTT, 0.5 μl of creatine phosphokinase [10 mg/ml in 50% glycerol; Roche], 0.5 μl RNasin [Promega], 1 μl S^35^-labeled methionine [ARC; >1000 Ci/mmol]).

Unless indicated otherwise, reactions were programmed with 1 μl of mRNA (0.1–1.0 μg) and incubated at room temperature for 1 hr. Further translation was stopped by addition of cycloheximide (1 mM). Microsomes (0.06 OD_280_) were then added and translocation allowed to proceed for an additional 30 min at room temperature. RNAs were digested with an RNase cocktail (Ambion). Finally, loading buffer was added and the translation products were analyzed by SDS-PAGE followed by phosphorimager analysis.

### Preperation of Translocation-Competent, ER-Derived Microsomes

Preperation of translocation-competent, ER-derived microsomes was performed as previously described (Brodsky, 2005). For more details see Experimental Procedures in the Supplemental Data.

### Immunoprecipitation and EndoH for In Vitro Reconstitution Experiments

Immunoprecipitation and EndoH for in vitro reconstitution experiments were performed as previously described (Stefanovic and Hegde, 2007). For more details see Experimental Procedures in the Supplemental Data.

## Supplemental Data

Document S1. Supplemental Experimental Procedures and Nine Figures
