# Supplementary material for: The GET Complex Mediates Insertion of Tail-Anchored Proteins into the ER Membrane
Source: Cell. 2008 Aug 22;134(4):634–45. doi: 10.1016/j.cell.2008.06.025 (PMC2572727; doi:10.1016/j.cell.2008.06.025)
Supplement: Document S1. Supplemental Experimental Procedures and Nine Figures [file mmc1.pdf]

Cell, Volume *134*

Supplemental Data

The GET Complex Mediates Insertion

of Tail-Anchored Proteins into the ER Membrane

Maya Schuldiner, Jutta Metz, Volker Schmid, Vladimir Denic, Magda Rakwalska, Hans Dieter Schmitt, Blanche Schwappach, and Jonathan S. Weissman

SUPPLEMENTAL EXPERIMENTAL PROCEDURES

Co-purification of 3HA-Get2 and Get1-4PC-HIS_{10}

p416MET25-Get1-8PC10His was constructed via an engineered NotI site coding for three alanines introduced to the 3'-end of the *GET1* open reading frame. In this construct the C-terminus of Get1 is fused to 8 PC epitopes (Roche) and a hexahistidine tag. Cloning of the *GET2* open reading frame into the vector BFGIII via a BamHI restriction site resulted in the N-terminal fusion of three HA epitopes to the Get2 protein. Δ*get1*/Δ*get2*/Δ*get3* yeast strain were transformed with the two plasmids (p416MET25-Get1-8PC10His and BFGIII-Get2) to over-express Get1-8PC10His and HA-Get2. The resulting strain was used for co-purification of both proteins. After preculturing in selective medium the yeast strain was grown in 2l YPD media to final OD_{600}=1.5-1.8 which resulted in approximately 20g of cells. Cells were collected by centrifugation for 10min at 4500g, washed once with water, shock frozen in liquid nitrogen and stored at -80°C.

For protein purification cells were thawed, washed once with cold lysis buffer (50mM Hepes/KOH pH 8.0, 200mM NaCl, 5mM $MgCl_2$, 0.5mM PMSF, 20mM β-mercaptoethanol and proteinase inhibitor mix containing 1.25mg/ml leupeptin, 0.75mg/ml antipain, 0.25mg/ml chymostatin and 5mg/ml pepstatin) and resuspended in 45ml cold lysis buffer. Yeast cells were opened using a microfluidizer (Microfluidics, Model 110) and debris was collected by centrifugation at 10,000g for 25min at 4°C, and supernatant was placed in ultracentrifugation tubes and centrifuged at 80,000g for 30min

at 4°C. The pellet obtained from the ultracentrifugation step contained membranes that were solubilized in 20ml extraction buffer (lysis buffer + 15mM imidazol, 50mM n-dodecyl-β-D-maltoside). Extraction of the membrane protein was performed for 1- 2h at 4°C with gentle shaking. Extracted protein was purified using Ni+ loaded sepharose beads (batch purification) at 4°C over night with gentle rotating. After binding, beads were washed twice with 10ml wash buffer (lysis buffer + 100mM imidazol, 0.2mM n-dodecyl-β-D-maltoside, 5% glycerin). After four washes the protein was eluted using 2 x 250ml elution buffer (lysis buffer + 1.5M imidazol 0.2mM n-dodecyl-β-D-maltoside, 5% glycerin). Purified protein was shock frozen in liquid nitrogen and stored at -80°C.

Yeast two-hybrid system

Constructs were cloned into vectors pGADT7 and pGBKT7 (BD-Clontech). The *GET3* bait construct was transformed into the yeast two-hybrid strain AH109 (BD-Clontech) and large-scale library transformations of a yeast genomic two-hybrid library (James et al., 1996). Primary co-transformants were screened on synthetic complete (SD) medium lacking tryptophane, leucine, and histidine. Positive colonies were streaked to SD medium lacking tryptophane, leucine, histidine, and adenine and subjected to a beta-galactosidase plate assay (overlay of the plates with the following solution containg 0.5% agarose: 0.5M sodium phosphate pH 7.0, 0.1% sodiumdodecylsulfate, 2% N,N' dimethylformamide, 0.2% 5-bromo-4-chloro-3-indolyl-beta-D-galactopyranoside). Library plasmid DNA from colonies displaying activity of all three reporter genes (*HIS3, ADE2, lacZ*) was rescued (Ausubel et al., 1997) and retransformed with the original bait construct and a GFP-containing control construct. Transformants were tested for the bait-specific activity of the *HIS3* and *ADE2* reporter genes and the corresponding plasmids were sequenced. For testing specific yeast two-hybrid constructs strain AH109 (Clontech) was transformed with the constructs indicated in the figure and plated on dropout medium lacking tryptophane and leucine to recover transformants. Individual colonies were grown in liquid culture and serial dilutions were spotted on dropout medium lacking tryptophane and leucine or lacking tryptophane, leucine, and histidine.

Fluorescence microscopy

For Figure 2B microscopy was performed using a Leica DM IRE2 microscope (Leica Microsystems, Wetzlar, Germany) controlled by OpenLab software (Improvision, Coventry, UK) with a 100x/1.4-0.7 HCX PL APO CS oil immersion objective. Images were captured by a Hamamatsu Orca-ER CCD camera (Hamamatsu Phototonics, Herrsching am Ammersee, Germany) with excitation at 470/40 nm and emission at 525/50 nm (GFP) or excitation at 510/40 nm and emission at 610 nm/longpass (tdRFP). For Figure 2C a DeltaVison restoration microscope equipped with a 100×/0.35-1.5 Uplan Apo objective and a GFP and RFP filter set (Chroma) was employed. Image stacks were collected with a Coolsnap HQ camera (Photometrics); the Z optical spacing was 0.2 µm. Raw images were deconvolved using the additive algorithm of Softworx software. All images shown are representative of several independent experiments. For live cell imaging yeast were incubated in synthetic complete medium at room temperature. Fixed yeast cells were mounted in ProLong Gold antifade reagent with DAPI (Invitrogen). Images were transferred to Adobe Photoshop CS2 for slight gamma adjustments.

For Figure 3 and 4 Light microscopy was performed in the UCSF Nikon Imaging Center with a Yokogawa CSU-22 spinning disc confocal on a Nikon TE2000 microscope. GFP was excited with the 488nm Ar-ion laser line and Cherry with the 568 nm Ar-Kr laser line. Images were recorded with a 100x / 1.4 NA Plan Apo objective on a Cascade II EMCCD. The sample magnification at the camera was 60nm/pixel. The microscope was controlled with Micro-Manager and ImageJ.

For the supplementary figures cells were analyzed by multiple wavelength fluorescence and visible light microscopy with a digital imaging capable Nikon TE200/300 inverted microscopy using an oil-immersed objective at X100 magnification.

Kar2 Secretion Assays

Cells were grown in YEPD at 30°C to mid-log phase, harvested, resuspended in fresh medium to OD_{600}=0.5 and incubated for 2h. Proteins were precipitated from 1.35ml of supernatants by addition of TCA to 10%. The pellet was washed with acetone, re-suspended in 35µl of 1x SDS-PAGE sample buffer and neutralized with 1mM Tris pH=9.4. Proteins were subjected to SDS–PAGE, transferred to a nitrocellulose membrane

and analyzed by immunoblotting using antisera against Kar2 (1:5000), (Kind gift from Peter Walter). Anti-rabbit secondary antibody conjugated to IRdye800 (Rockland) (used at 1:10,000) was detected using Odyssey (Li-COR) fluorescent scanner and software.

Preparation of Microsomes

Membranes were prepared from cells grown to OD_{600} 1-2 in YPD by spheroplast lysis. Briefly, cells (~1000-2000 OD_{600}) were washed with water and treated with 40ml of 100mM Tris-HCl pH9.5 10mM DTT for 10minutes at room temperature. Following centrifugation, the cell pellet was resuspended in 20ml spheroplasting buffer (0.7M sorbitol, 75% YP media, 0.5% glucose, 10mM Tris-HCl pH7.4 containing 10-20U/OD_{600} of homemade recombinant lyticase) and incubated at 30°C until >90% spheroplasting efficiency was achieved. 20ml of the spheroplast suspension was collected through a 20ml cushion (0.8M sucrose, 1.5% Ficoll 400, 20mM HEPES-KOH pH7.4) by spinning in an HB-4 rotor at 6,000rpm for 10minutes. The spheroplast pellet was resuspended in 15ml of lysis buffer (0.1M sorbitol, 50mM KOAc, 2mM EDTA, 20mM HEPES-KOH pH7.4, 1mM DTT, complete protease inhibitors from Roche) and dounced 25 times with a glass homogenizer. The lysate was spun through a 15ml cushion (1.0M sucrose, 50mM KOAc, 20mM HEPES-KOH pH7.4, 1mM DTT) in an HB-4 rotor at 6,500rpm for 10minutes. The supernatant was collected and spun in a Ti50.2 rotor at 16,700rpm for 20min. The crude microsome pellet was washed in B88 (20mM HEPES-KOH pH6.8, 150mM KOAc, 250mM sorbitol, 5mM MgOAc) and the Ti50.2 spin repeated. The washed microsomes were resuspended in B88 at a final OD_{280} ~40-50.

Co-Immunoprecipitation

Yeast cells were grown to an OD_{600}=0.5 in the appropriate dropout medium lacking leucine and uracil and containing 2% glucose as the carbon source. 150 OD_{600} units were harvested and broken in 500μl breaking buffer (20mM HEPES pH 7.4, 100mM KCl, 2×Complete protease inhibitor cocktail; Roche) using glass beads. The resulting extract was diluted 1:1 with immunoprecipitation buffer (50mM Tris-HCl pH 7.6, 150mM NaCl, 1mM EDTA, and 1% NP-40, complemented with Complete protease inhibitor) and incubated with 5μg mouse monoclonal anti-HA (HA.11, Covance) and protein G-

sepharose (Amersham) overnight, washed three times in immunoprecipitation buffer and several times in PBS, and eluted in SDS-PAGE loading buffer without reducing agent at 37°C.

Whole Cell Extracts and Immunoblotting

Cells were grown in SD dropout medium at 30°C to mid-log phase, then transferred to a 1.5ml reaction tube and centrifuged for 5min at 4,000 rpm. The pellet was resuspended in 200µl of 0.1M NaOH. After a 10 minute incubation at room temperature the density of the cell suspension was assayed by OD_{600} measurement. The sample was centrifuged for 1min at 13,000rpm and the pellet was lysed in SDS-PAGE sample buffer. 10µl of each sample (equivalent of 0.01 OD_{600} unit per lane) was resolved by SDS-PAGE.

Yeast extracts and immunoprecipitates were separated by SDS-PAGE using 10% gels and transferred to nitrocellulose. Blots were blocked in TBS containing 5% milk powder and 0.02% NP-40. Primary antibodies (anti-HA mouse monoclonal HA.11, Covance, 1µg/ml; anti-PC mouse monoclonal HPC4, Roche, 0.25µg/ml; anti-Pep12 mouse monoclonal, 0.5µg/ml (Invitrogen); anti-Arr4p guinea pig serum (Metz et al., 2006), 1:4,000; affinity-purified rabbit anti-Sed5, 1:1,000 (Kind gift from Dieter Gallwitz); anti-Emp47 rabbit serum, 1:1,000 (Kind gift from Stephan Schröder-Köhne and Howard Riezman); anti-Sec61 rabbit serum, 1:25,000 (Kind gift from Matthias Seedorf); affinity-purified rabbit anti- Rer1, 1:500; affinity-purified rabbit anti-GFP, 1:4,000 (Kind gift of Dirk Görlich) and secondary antibodies (HRP-conjugated anti-mouse, anti-guinea pig, and anti-rabbit antibodies, Jackson, 1:1,000) were diluted in TBS-blocking solution. Washes were in TBS-blocking solution and then in TBS, 0.02% NP-40. Detection was performed using the ECL system (Amersham) or an Odyssey (Li-COR) fluorescent scanner.

Subcellular Fractionation on Sucrose Gradients

Separation of organelles was performed as described {Nass, 1998 #73}. Briefly, cells were grown to mid-logarithmic phase, converted to spheroblasts and lysed by douncing in a hypotonic buffer (0.3M sorbitol, 50mM triethanol amine pH 8.9). The homogenate was layered on freshly prepared ten-step sucrose gradients and centrifuged for 12h at

23,500rpm in an SW28 rotor (Beckman). Membranes from the different fractions were pelleted by centrifugation (SW28, 30minutes at 23,500rpm). 10µg of membrane protein was loaded per fraction and resolved by SDS-PAGE.

Triton-Solubility Assay Employing Subcellular Fractions

10µg protein of pellets obtained from the dense sucrose fractions 8 and 9 (46% and 50%) were incubated for 10minutes in 20µl 20mM HEPES, 1% Triton-X100 at 30°C. After a 15min 16,000rcf centrifugation step, 4µl 5xSDS-PAGE sample buffer and 100mM DTT were added to the supernatant. The pellet was resuspended in 25µl 1xSDS-PAGE sample buffer including 100mM DTT. 15µl of each pellet and supernatant were subjected to SDS-PAGE, transferred to a nitrocellulose membrane and analyzed by immunoblotting using antiserum against Get3.

Protease protection for in vitro translocation assays

Following translocation, the reactions were treated with 0.25mg/ml Proteinase K (Worthington Biochemical Company) in either the presence or the absence of 1% Triton X-100 on ice for 30min. PMSF was then added (10mM) followed by further incubation on ice for 5min. Finally, boiling loading buffer was added and the samples were kept boiling for 5min to ensure that Proteinase K is irreversibly inactivated.

SUPPLEMENTAL FIGURES:

Schuldiner et al., Figure S1

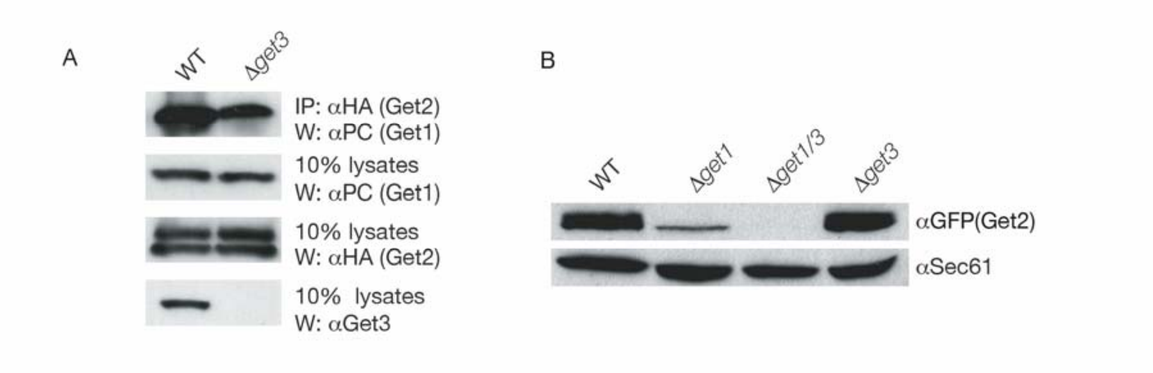

(A) Western blots of Co-Immunopercipitation experiments performed on either control (WT) or Δget3 strains expressing tagged Get2-HA and Get1-PC. 10% input lysates are shown to demonstrate the constant levels of Get proteins in the cells.

(B) Western blots immunostained against GFP in yeast expressing Get2-GFP demonstrate the reduced levels of Get2 in Δget1 strains.

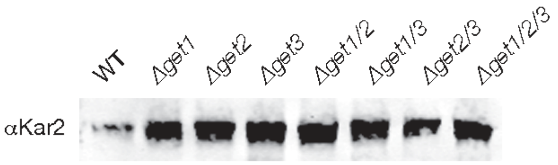

Western blots of secreted proteins immunostained against the ER resident chaperone Kar2. Assay for Kar2 secretion was performed on all single, double and triple mutant combinations of *get* gene deletions and analyzed relative to a control strain (WT).

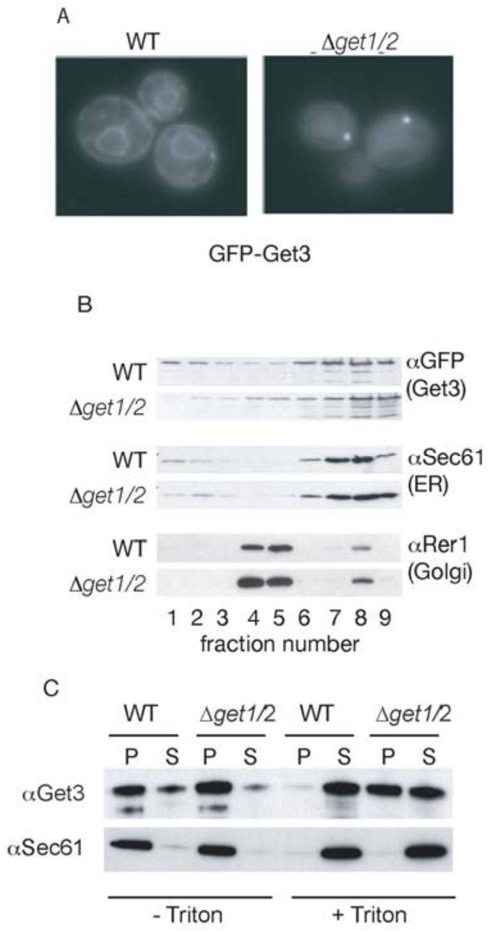

(A) Fluorescence microscopy demonstrating a shift in the sub-cellular localization of Get3-GFP from ER in control (WT) strains to large puncta in Δget1/Δget2 strains.

(B) Sucrose gradient sub-cellular fractionation experiments. Shown are the fractions from light (1) to heavy (9) in control (WT) and Δget1/Δget2 strains. Western blots were probed against GFP to detect the presence of GFP-Get3 and against representative endogenous proteins from the ER (Sec61) and Golgi (Rer1) as control.

(C) Get3 forms detergent insoluble aggregates in the absence of Get1/2. The heavy fractions from the sucrose gradient shown in panel B, were treated with 1% Triton-X100 and subjected to centrifugation. The pellet (P) and supernatant (S) were then probed with an anti-Get3 antibody.

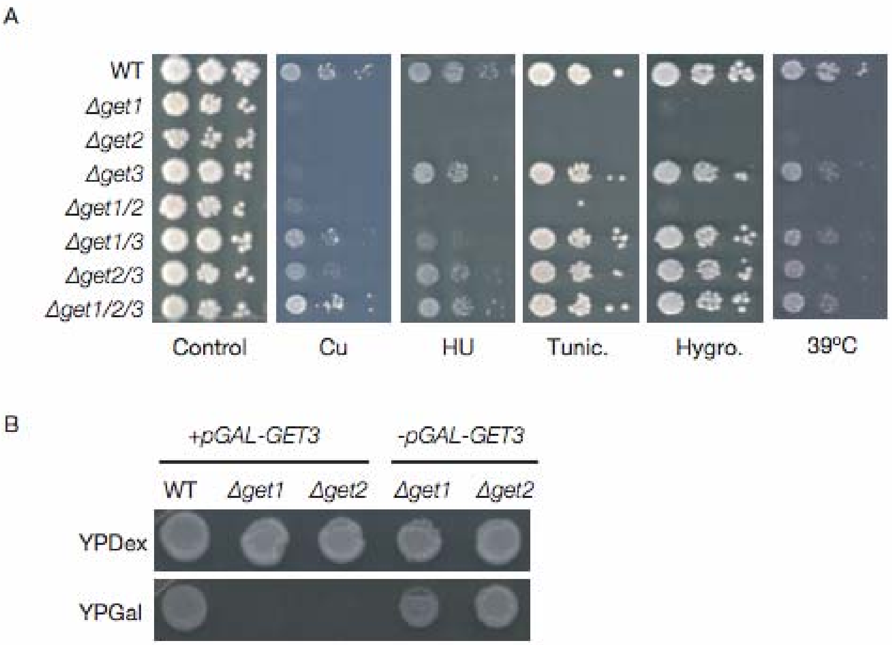

(A) Serial dilutions of *get* deletion mutants and a control strain (WT) on 5 different plate assays that reveal a wide range of sensitivities: SD+CuSO4 (Cu), YPD+ hydroxyurea (HU), YPD + tunicamycin (Tunic.), YPD+ hygromycin (Hygro.) and YPD incubated at 39°C (39°C). Note that the deletion of get3 improved the growth of Δget1 and Δget2 strains.
(B) Growth of control (WT), Δget1 and Δget2 strains expressing GET3 under regulation of a galactose-inducible promoter (pGAL-GET3). Growth was assayed on either medium containing galactose (YPGal) to induce expression or dextrose (YPDex) to repress expression. Over-expression of GET3 had no effect on growth of WT cells, however, in a Δget1 or Δget2 background it was toxic.

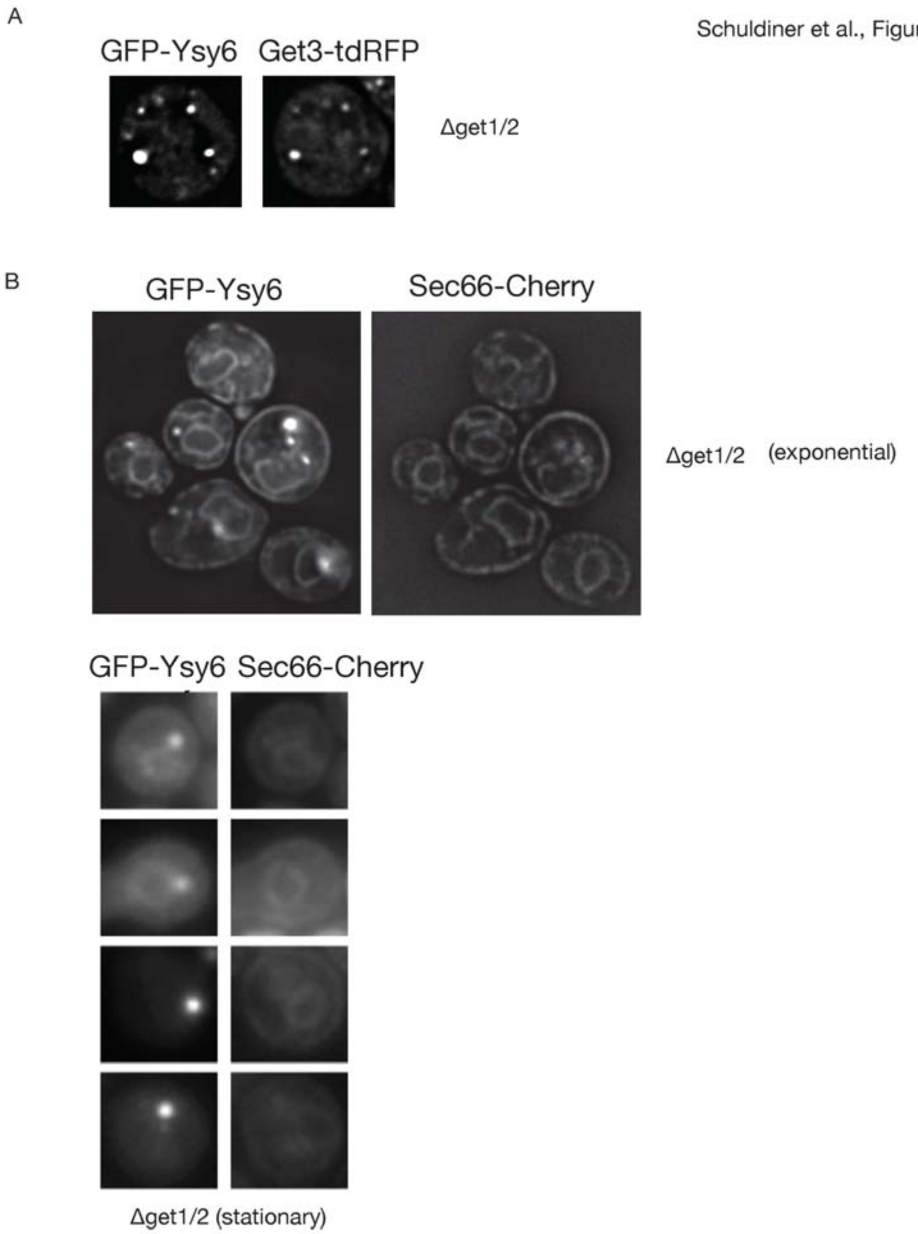

(A) Fluorescence microscopy demonstrating the colocalization of Ysy6 and Get3 within punctate cytosolic aggregates in Δget1/2 mutants.
(B) Fluorescence microscopy demonstrating the normal structure of ER membranes in Δget1/Δget2 mutants in either exponential or stationary growth phase. expressing GFP-Ysy6. Sub-cellular localization of a control ER protein, Sec66, does not change, demonstrating that ER morphology is intact in Δget1/Δget2

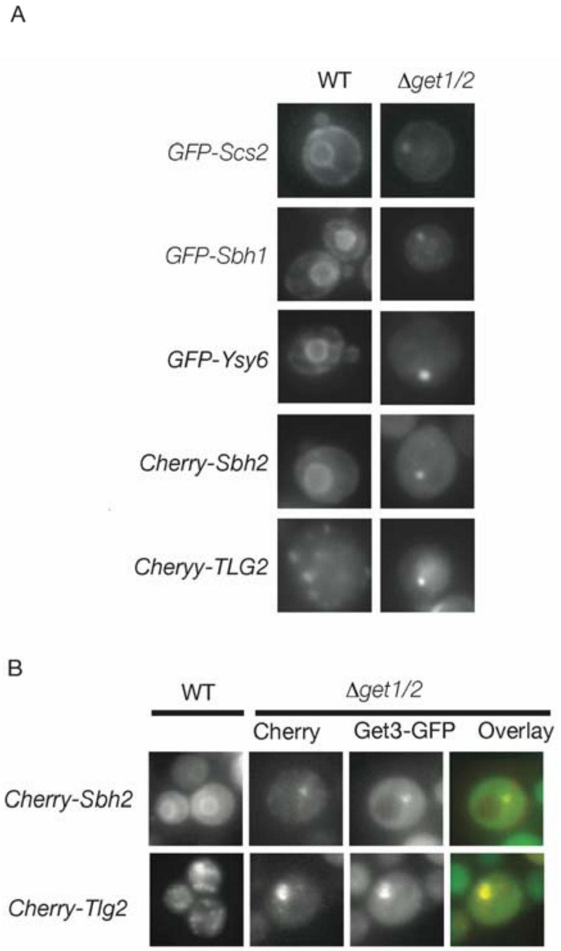

(A) Fluorescent microscopy of control (WT) and Δget1/2 strains in stationary phase expressing a broad variety of TA proteins. Scs2, Sbh1 and Ysy6 tagged with N terminal GFP were expressed from a Gal inducible promoter. Sbh2 and Tlg2 were tagged with an N terminal Cherry and expressed from a plasmid under the constitutive Tef2 promoter.
(B) Co-localization with a fluorescent marked Get3-GFP demonstrates the presence of both Get3 and TA proteins in the same cytosolic puncta.

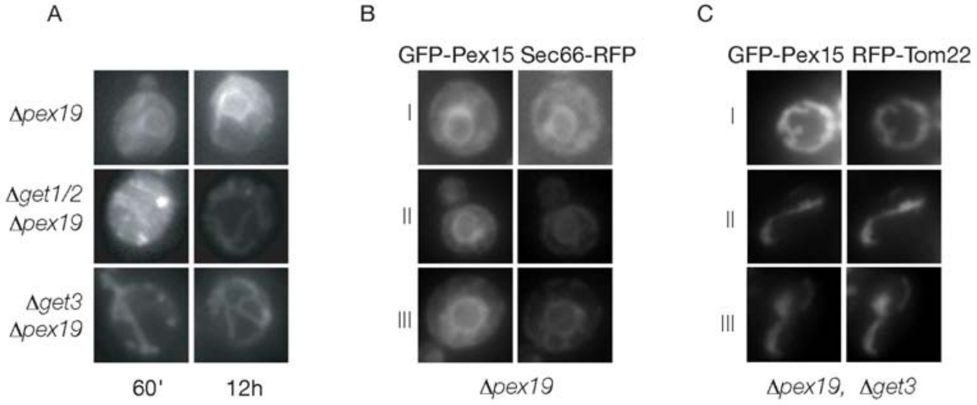

(A) Fluorescence microscopy demonstrating that mis-localization of GFP-Pex15 in Δget1/Δget2 mutants is not dependant on its subsequent trafficking from the ER to the peroxisomes. GFP-Pex15 localization was followed after induction from a Gal inducible promoter and its subcellular localization was viewed on the background of a mutation which reduces peroxisomal biogenesis (Δpex19).

(B) Three examples of fluorescent microscopy demonstrating the co-localization of the ER protein, Sec66, with the TA protein Pex15 in a Δpex19 mutant 1 hour after induction of GFP-Pex15.

(C) Three examples of fluorescent microscopy demonstrating the co-localization of the mitochondrial protein Tom22 with Pex15 in a Δpex19/Δget3 mutant 1 hour after induction of GFP-Pex15.

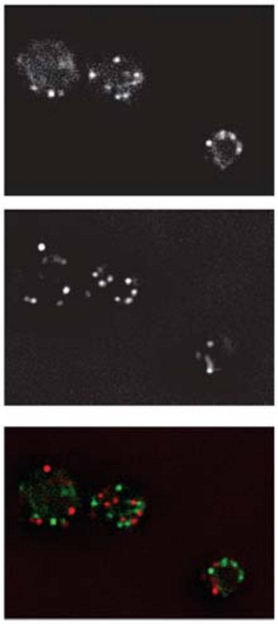

Fluorescence microscopy demonstrating the mislocalization of GFP-Pex15 in the Δget1/2 mutants despite the normal structure of the peroxisome. GFP-Pex15 staining was followed after induction from a Gal inducible promoter and its subcellular localizationis appears distinct from the peroxisomal marker Pex3-Cherry.

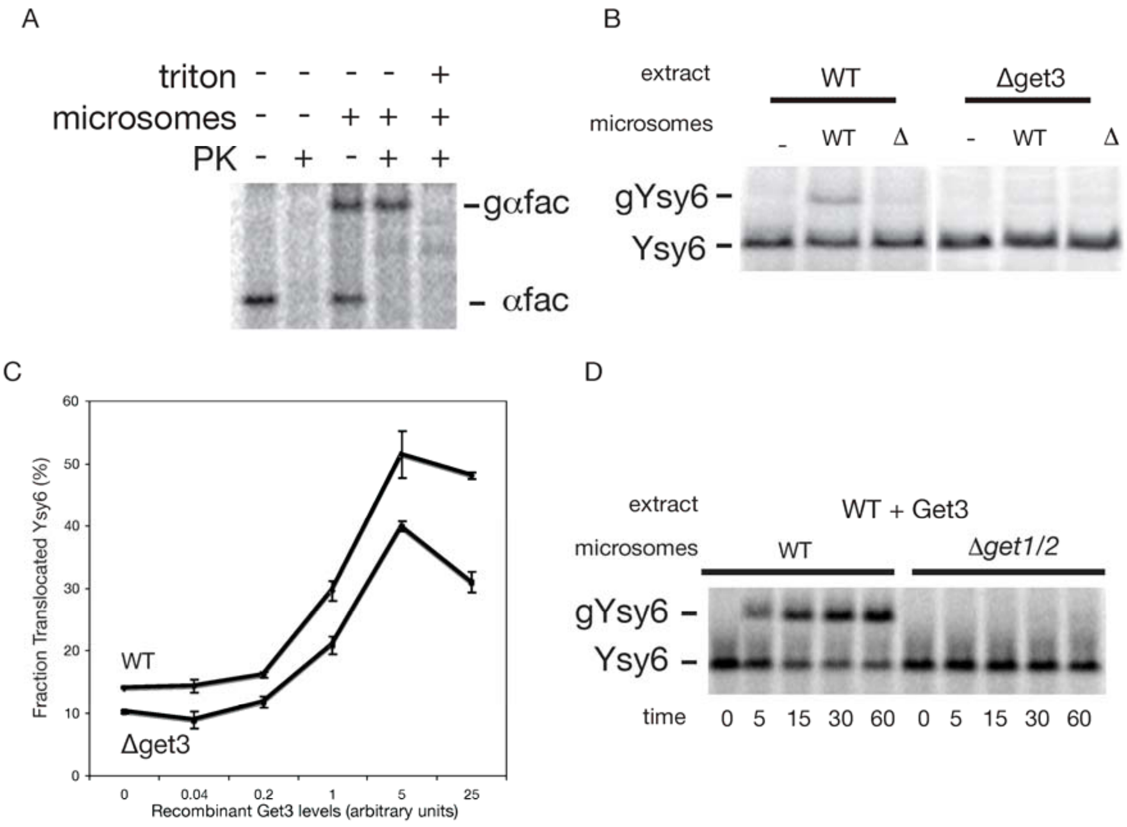

(A) Autoradiograph of in vitro translated, ^{35}S Methionine labeled, alpha factor following incubation in the presence of microsomes derived from wild type (WT) cells. Following translocation samples were treated with either Proteinase K (PK) alone or in the presence of detergent (triton). The position of untranslocated prepro α-factor (αfac) and glycosylated, translocated proα-factor (gαfac) are indicated. The differential protection of glycosylated alpha factor demonstrates that the microsomes are intact and capable of supporting efficient translocation and glycosylation of proteins.

(B) Autoradiograph of ^{35}S Methionine labeled Ysy6 translated in extracts derived from either WT or Δget3 strains. Translated protein was incubated with microsomes derived from either wild type (WT) or *Δget1/2* strains. The position of untranslocated Ysy6 as well as glycosylated translocated Ysy6 (gYsy6) are indicated demonstrating the requirement for Get3 in the extract for allowing efficient translocation of Ysy6 into microsomes in a Get1/2 dependant manner

(C) Dose dependence of Ysy6 translocation on addition of recombinant Get3 to either WT or *Δget3* derived translation extracts. WT microsomes were added following translation and the amount of glycosylated Ysy6 relative to total Ysy6 was calculated. Results from three independent experiments are shown. Note, we observe some batch-to-batch differences in the translocation efficiency of unsupplemented extracts (e.g. we see somewhat more efficient Get3-dependent translocation in panel B). This likely reflects the fact that Get3 is limiting in the diluted extracts. Indeed, in all cases we see robust Ysy6 insertion in extracts supplemented with Get3 and this insertion is completely dependent on Get1/Get2. Moreover, the dose dependency for the Get3 stimulation is highly similar for WT and Δget3 extracts.

(D) Autoradiograph of ^{35}S Methionine labeled Ysy6 following translation in WT cytosol supplemented with optimal levels of Get3. Translocation was terminated the indicated times following addition of microsomes derived from either wild type (WT) or *Δget1/2* strains. The position of untranslocated Ysy6 as well as glycosylated translocated Ysy6 (gYsy6) are indicated, demonstrating that efficient Get3 mediated translocation is strictly dependant on the presence of Get1/2 in the microsomes even during extended incuabtion times (60 minutes vs the standard 30 minute incubation time).
